# Microenvironmental Drivers of Bone Disease in Multiple Myeloma: Oxidative Stress, Sterile Inflammation, Autophagy–Lysosomal Remodeling, and the Iron–Lipid Peroxidation Axis

**DOI:** 10.3390/biom16050710

**Published:** 2026-05-12

**Authors:** Maria Elisa Nasso, Adele Bottaro, Manlio Fazio, Fabio Stagno, Sebastiano Gangemi, Alessandro Allegra

**Affiliations:** 1 Division of Hematology, Department of Human Pathology in Adulthood and Childhood “Gaetano Barresi”, University of Messina, Via Consolare Valeria 1, 98125 Messina, Italy; mariaelisanasso@gmail.com (M.E.N.); adelebottarp15@gmail.com (A.B.); manliofazio@hotmail.it (M.F.); stagnof@unime.it (F.S.); 2 Allergy and Clinical Immunology Unit, Department of Clinical and Experimental Medicine, University of Messina, Via Consolare Valeria, 98125 Messina, Italy; gangemis@unime.it

**Keywords:** oxidative stress signaling, sterile inflammation axis, autophagy–lysosomal remodeling, iron–lipid peroxidation network, ferroptosis susceptibility, osteoclastogenic microenvironment, bone marrow niche reprogramming, nox4–nrf2 modulation, DAMP–RAGE/TLR pathways, ferroportin-1 dysregulation

## Abstract

Multiple myeloma profoundly remodels the bone marrow microenvironment, causing osteolytic bone disease through a persistent uncoupling of bone resorption and formation. Beyond the canonical roles of the receptor activator of nuclear factor kappa-B ligand/receptor activator of nuclear factor kappa-B/osteoprotegerin triad and Wnt antagonism, three interdependent stress programs orchestrate the osteolytic niche. These include oxidative stress driven by mitochondrial and nicotinamide adenine dinucleotide phosphate oxidase-derived reactive oxygen species; sterile inflammation sustained by damage-associated molecular patterns, pattern-recognition receptors, and pro-inflammatory cytokines; and autophagy–lysosomal remodeling governed by transcription factor EB and the coordinated lysosomal expression and regulation network. These axes intersect with iron handling and lipid peroxidation to regulate sensitivity to ferroptotic cell death, thereby shaping osteoclast priming, osteoblast suppression, and matrix turnover. Building on these mechanistic insights, we outline a translational framework that aligns standardized bone turnover markers of formation and resorption with composite panels of oxidative and nitrosative stress. This framework also integrates modern imaging to capture structural injury and metabolically active marrow disease. We further propose a therapeutic roadmap layered on antiresorptive foundations that targets selective inhibition of nicotinamide adenine dinucleotide phosphate oxidase 4 and calibrated modulation of nuclear factor erythroid 2–related factor 2, disrupts damage-associated molecular pattern and cytokine circuits, and applies lineage- and timing-specific tuning of autophagy together with restoration of ferroportin-1 or iron chelation. This integrated strategy is designed to recouple bone remodeling and improve clinically meaningful skeletal outcomes in multiple myeloma.

## 1. Introduction

### 1.1. Canonical Mechanisms of Bone Remodelling Imbalance

Multiple myeloma is a malignant plasma-cell disorder that remodels the bone-marrow ecosystem and culminates in osteolytic bone disease in most patients, producing pain, pathological fractures, and other skeletal-related events despite significant gains in anti-myeloma therapy [1,2,3]. The canonical explanatory frame remains an uncoupling of bone remodeling: osteoclast formation and function are amplified while osteoblast differentiation and bone formation are suppressed. At the center of this imbalance is the RANKL/RANK/OPG system—excessive receptor activator of nuclear factor κB ligand (RANKL) signaling on osteoclast precursors, coupled with reduced osteoprotegerin (OPG), accelerates osteoclastogenesis and resorption [4]. In parallel, Wnt antagonism—particularly myeloma-derived Dickkopf-1 (DKK1) and osteocyte-derived sclerostin—constrains osteoblastogenesis and mineral deposition [3,5].

### 1.2. Stress-Adaption Programs Shaping the Osteolytic Niche

This two-pronged perturbation, however, insufficiently explains the persistence and breadth of skeletal pathology. Accumulating evidence places microenvironmental stress-adaptation programs as higher-order regulators that organize the osteolytic niche across cellular compartments. Three interdependent programs are especially salient. First, oxidative stress driven by mitochondrial and NOX4-derived reactive oxygen species (ROS) amplifies osteoclast differentiation and activity through NF-κB/MAPK and CaMK–CREB→NFATc1 signaling. In parallel, it shifts mesenchymal and osteocytic outputs toward RANKL expression and away from osteoblast commitment [6,7]. Second, sterile inflammation—fueled by damage-associated molecular patterns (DAMPs) such as High Mobility Group Box 1 (HMGB1) acting through Receptor for Advanced Glycation End-products (RAGE) and Toll-Like Receptors (TLR) circuits, and by cytokines including interleukin-1β, tumor necrosis factor-α, and interleukin-6—maintains osteoclast priming and couples tumor survival signals to bone resorption [8,9].

Third, autophagy–lysosomal remodeling, transcriptionally governed by Transcription Factor EB (TFEB) and the Coordinated Lysosomal Expression And Regulation (CLEAR) network, calibrates catabolic capacity in both osteoclasts and myeloma cells; core autogaphy-related proteins, (ATG) proteins, are required for polarized lysosomal secretion and ruffled-border formation, whereas proteasome inhibition in myeloma elicits a terminal unfolded protein response (UPR) that intersects with autophagy to define therapeutic sensitivity [10,11].

These stress programs are tightly interconnected. ROS amplify inflammatory transcription and RANKL induction, while DAMPs and cytokines further increase NOX activity. Autophagy, in turn, preserves proteostasis under persistent oxidative and inflammatory stress [12].

Conceptually, these pathways operate within a hierarchical framework rather than as equivalent parallel processes. Canonical disruptions of bone remodeling—namely RANKL/OPG imbalance and Wnt antagonism—represent the primary initiating layer of uncoupling. Superimposed upon this foundation, oxidative stress, sterile inflammation, and autophagy–lysosomal remodeling function as higher-order stress-adaptation programs that amplify, stabilize, and perpetuate osteolytic dominance over time. Ferroptosis susceptibility, in turn, emerges downstream as a context-dependent consequence of sustained redox imbalance, iron handling, and lipid remodeling rather than as an independent upstream driver.

### 1.3. Osteocyte Dysfunction and Microarchitectural Deterioration

An emergent iron–lipid peroxidation dimension further shapes osteoclast biology and therapy resistance. In macrophages and osteoclast precursors, ferroportin-1 (FPN1) downregulation promotes intracellular iron retention and favors osteoclastogenesis; restoration of FPN1 or iron chelation constrains osteoclast differentiation in myeloma co-cultures [13]. Concurrently, the accumulation of phospholipid hydroperoxides—particularly oxidized phosphatidylethanolamines—under conditions of glutathione peroxidase-4 (GPX4) insufficiency and ACSL4-driven enrichment of peroxidation-prone acyl chains defines susceptibility to ferroptosis, a regulated, iron-dependent cell-death program [14,15]. These processes establish a redox–lipid rheostat that can influence both tumor cells and bone-resorbing lineages, raising prospects for selective, cell-type-aware interventions.

The aim of this review is to provide an in-depth analysis of the role of uncoupling mechanisms in the pathogenesis of bone disease in patients with multiple myeloma and to propose an evidence-based framework for its clinical management.

This narrative review is based on a comprehensive appraisal of the peer-reviewed literature indexed in PubMed up to January 2026. We prioritized original experimental studies, translational investigations, and high-quality reviews addressing bone marrow microenvironmental alterations in MM, with particular emphasis on oxidative stress, sterile inflammation, autophagy–lysosomal remodeling, iron metabolism, and ferroptosis-related pathways.

Priority was given to studies providing mechanistic insight supported by in vitro, in vivo, or patient-derived data, as well as to seminal contributions that have shaped current understanding of myeloma-associated bone disease. When available, clinical, and translational evidence was highlighted separately from preclinical findings. Given the heterogeneity of study designs and the evolving nature of the field, a narrative and integrative approach was deliberately adopted to contextualize emerging pathways within established models of bone remodeling uncoupling.

During manuscript preparation, Microsoft 365 Copilot (GPT-5 chat model) was used solely as an auxiliary tool for language refinement, figure conceptualization, and organizational support. All scientific content was independently produced, reviewed, and approved by the authors.

## 2. From Coupled to Uncoupled Remodeling in MM

### 2.1. Drivers of Uncoupling in Myeloma Microenvironment

Osteocytes—by coordinating RANKL/sclerostin outputs and performing perilacunar–canalicular remodeling—translate mechanical and inflammatory cues into remodeling instructions. In myeloma, osteocyte dysfunction and altered perilacunar remodeling have been linked to cortical thinning and trabecular perforation, reinforcing the osteolytic phenotype [16,17]. More broadly, stromal, and immune components of the marrow microenvironment provide survival factors and inflammatory signals that consolidate osteoclast priming and tether bone resorption to myeloma fitness [18,19,20].

Bone remodeling relies on a choreography among osteoclasts (OCs), osteoblasts (OBs), and osteocytes, coordinated by stromal, vascular, and immune lineages. In MM, myeloma and stromal cells upregulate RANKL and reduce OPG, accelerating OC differentiation. In parallel, DKK1 and sclerostin suppress osteoblastogenesis, consolidating net resorption and impairing microarchitectural integrity. Cytokine circuits integrate tumor signals with innate sensors to cement the osteolytic phenotype [18,20].

### 2.2. Derailment of Coupling Signals—Osteocyte Mechanics, Redox Tone and Perilacunar Remodeling: Cytokine-Danger Signal Integration

The observations reported above have several translational corollaries that clarify why durable control of bone remodeling in multiple myeloma requires multi-layered therapeutic strategies.

Because uncoupling is sustained by networked stromal–immune drivers, therapeutic control demands layered strategies that simultaneously blunt RANKL-driven osteoclastogenesis and relieve Wnt antagonism while decompressing cytokine pressure [21]. Antiresorptives remain foundational yet insufficient to restore formation; adjunctive targeting of IL-6/TNF-α/IL-1β circuits and the HMGB1–RAGE axis, together with osteoanabolic relief from DKK1/sclerostin blockade, represents a rational path to recouple remodeling in MM [22].

The interplay between osteocyte mechanics, redox tone, and microdamage accumulation provides a critical framework for understanding how osteocytic dysfunction perpetuates structural fragility in multiple myeloma. In MM this interplay perturbs osteocytic mechanotransduction, and reduces anabolic signaling to surface osteoblasts, thereby propagating bone alterations [23]. The resulting deterioration in local material properties perpetuates microdamage accumulation, which further stimulates osteoclast recruitment and activity, creating a self-reinforcing loop of resorptive dominance [24].

The integration of cytokine signaling with danger-associated molecular pathways shapes an osteoimmune environment that permits sustained osteoclast activation. Pro-inflammatory cytokines such as IL-1β, TNF-α, and IL-6 cooperate with danger-associated molecular patterns including HMGB1 to enhance osteoclastogenic competence. This “licensing” effect reflects a reduced activation threshold in osteoclast precursors, driven by RAGE- and TLR-dependent amplification of NF-κB and MAPK signaling, which synergizes with RANKL to sustain NFATc1 induction and osteoclast differentiation while concomitantly suppressing osteoblast lineage commitment [25,26,27].

In physiological bone remodeling, specific coupling signals coordinate osteoclast and osteoblast activities, but in multiple myeloma these regulatory pathways become profoundly derailed. In healthy bone, bidirectional coupling hinges on osteoclast-derived cues (e.g., S1P, ephrinB2) that stimulate osteoblast recruitment and maturation, and on osteoblast-produced factors that restrict excessive resorption, ensuring spatiotemporal fidelity of remodeling units [28]. In multiple myeloma, this choreography is progressively dismantled as tumor–stromal interactions skew lineage outputs toward sustained resorption and impaired formation. BMSCs and osteocytes—conditioned by myeloma cells— modify osteoclast commitment and survival [29].

### 2.3. Potential Diagnostic Assessments of Cytokine-Induced Uncoupling and Possible Therapeutic Interventions

To translate mechanistic insights into measurable outcomes, it is essential to operationalize redox-informed endpoints that capture early shifts in bone remodeling dynamics. From a translational vantage, coupling standardized bone turnover markers (serum Procollagen Type I N-Terminal Propeptide (PINP/CTX) with oxidative/nitrosative composites (AOPP, AGEs, S-nitrosylated proteins) provides early, mechanism-sensitive readouts of niche reprogramming during redox-targeted therapy [30]. Integrating these biomarker shifts with IMWG-endorsed imaging (LD-WBCT, WB-MRI, FDG-PET/CT) refines discrimination between mere antiresorptive stabilization and true anabolic recovery within metabolically active disease, enabling adaptive trial designs in MM bone disease [31,32].

Because redox and signaling pathways operate in a highly context-specific manner, therapeutic interventions must be carefully calibrated to preserve physiological remodeling while disrupting pathological drivers. While NOX4 generally enforces a pro-resorptive program, cell-context matters: in defined settings NOX4 can support osteoblast differentiation through TGF-β signaling, underscoring the need for calibrated interventions that spare physiological redox signaling required for bone formation [33]. Consequently, selective NOX4 inhibition and tunable activation of the cytoprotective NRF2 pathway are attractive levers to preserve host defense and osteolineage competence [34,35].

## 3. ROS-Dependent Osteoclastogenic Pathways—Role of Ferroptosis

Understanding the sources and spatial topology of ROS signaling within the marrow niche is essential for deciphering how oxidative stress shapes osteoclast and stromal cell behavior in myeloma. Reactive oxygen species originate from mitochondrial electron leakage and NADPH oxidases, with NOX4 emerging as a dominant source in osteoclast-lineage cells exposed to myeloma-conditioned cues [33]. Mitochondrial and NOX4-derived ROS converge on NF-κB/MAPK and CaMK–CREB→NFATc1 cascades, accelerating osteoclast differentiation and cytoskeletal organization. This signaling convergence ultimately enhances ruffled-border formation and resorptive activity [33,36,37].

Current evidence supporting the role for ferroptosis in the myeloma bone microenvironment is largely derived from preclinical models and mechanistic studies conducted in vitro or in vivo under experimentally controlled conditions. While these data provide compelling biological plausibility, they do not yet establish ferroptosis as a dominant or universal mechanism driving bone destruction in patients with multiple myeloma.

Within the cellular environment, multiple ferroptosis-defense nodes are activated in response to the prevailing oxidative tone. At the plasma membrane, FSP1 (AIFM2) regenerates ubiquinone/CoQ10 to ubiquinol, acting in parallel with GPX4 to quench lipid peroxyl radicals; N-myristoylation-dependent membrane targeting is essential for its anti-ferroptotic function. Within mitochondria, DHODH complements mitochondrial GPX4 by regenerating ubiquinol at the inner membrane; genetic or pharmacologic DHODH inhibition selectively sensitizes GPX4^low^ tumors to ferroptosis and synergizes with inducers, although context-dependent off-target effects demand careful dose selection [38,39].

In this redox-conditioned microenvironment, the cellular response extends beyond lipid-peroxidation control mechanisms and begins to intersect with broader inflammatory and osteolytic programs. Sustained oxidative pressure not only activates ferroptosis-defense mechanisms but also reshapes the immunometabolic state of myeloid and stromal cells, lowering their threshold for inflammatory activation.

As antioxidant nodes such as FSP1 and DHODH work to contain lethal lipid peroxidation, residual ROS flux simultaneously functions as a potent signaling currency, propagating stress cues across the bone marrow niche. This redox spillover primes innate immune sensors, alters stromal homeostasis, and sets the stage for inflammasome-dependent amplification loops that decisively shift the balance from controlled tissue maintenance toward pathological osteoclastogenic activation.

In the myeloma setting, the crosstalk between ROS and the NLRP3 inflammasome becomes increasingly pronounced. Oxidative stress primes and activates the NLRP3 inflammasome in myeloid cells, licensing caspase-1–dependent maturation of IL-18 that further reinforces RANKL expression and osteoclast recruitment. In multiple myeloma, uptake of β2-microglobulin by tumor-associated macrophages provokes lysosomal damage and NLRP3 activation, accelerating inflammatory signaling that fuels disease progression and osteolysis; experimental inhibition of NLRP3 mitigates tumor growth and bone destruction in preclinical models [40].

Within this framework, even the aforementioned cytokines appear to exert their effects through mechanistically distinct signaling pathways. In fact, within the myeloma niche, IL-6 trans-signaling functions as a potent amplifier of redox imbalance. Beyond classic IL-6 signaling through membrane IL-6R, the trans-signaling route—mediated by soluble IL-6R engaging gp130 on target cells—broadens the spectrum of marrow elements responsive to IL-6 and potentiates osteoclastogenic programs under oxidative load. In osteocytes and differentiated osteoblasts, IL-6 trans-signaling selectively cooperates with ADAM17-dependent shedding of IL-6R, thereby coupling cytokine tone to resorptive bias; conversely, indiscriminate systemic blockade can impair skeletal homeostasis, arguing for calibrated modulation [41,42].

**Figure 1:**
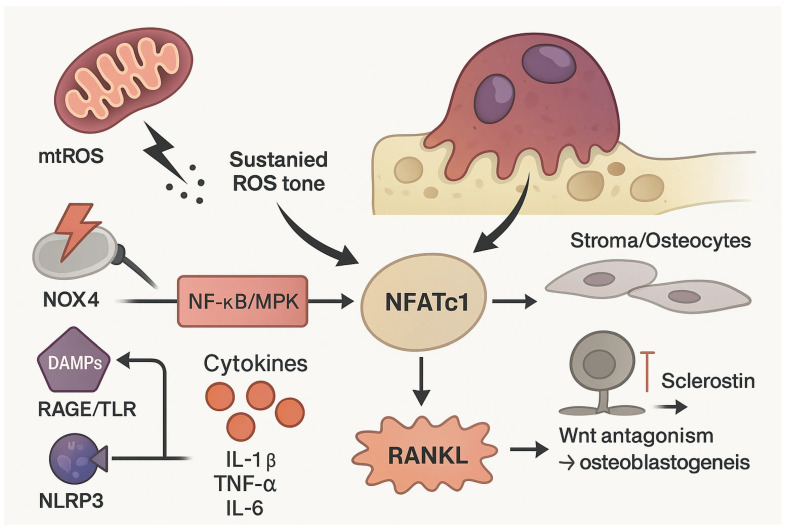
Oxidative stress and sterile inflammatory cues converge to amplify osteoclast differentiation and function in the myeloma bone marrow niche. Reactive oxygen species (ROS) derived from mitochondrial dysfunction and NADPH oxidase 4 (NOX4) activate NF-κB and MAPK pathways in osteoclast precursors and cooperate with CaMK–CREB signaling to sustain NFATc1 induction downstream of RANKL. In parallel, danger-associated molecular patterns (DAMPs), particularly HMGB1, engage RAGE and Toll-like receptors (TLRs), lowering the activation threshold of osteoclast precursors by reinforcing NF-κB/MAPK signaling. Pro-inflammatory cytokines (IL-1β, TNF-α, IL-6) further potentiate this response by increasing RANKL expression in stromal cells and osteocytes while suppressing Wnt-dependent osteoblast differentiation. Collectively, these inputs “license” osteoclastogenesis by stabilizing NFATc1-driven transcriptional programs and shifting remodeling balance toward persistent bone resorption.

Therapeutically, cytokine and selective NOX4 targeting and calibrated NRF2 modulation are rational strategies to reduce pathological ROS while preserving physiological redox signaling and immune competence. See Figure 1.

## 4. Sterile Inflammation and Osteoimmunology

Extended osteoimmunology sources integrate HMGB1–RAGE/TLR circuits with cytokine milieus and inflammasome signaling that sustain osteoclastogenic tone.

The myeloma marrow exhibits sterile inflammation, where DAMPs engage pattern-recognition receptors to sustain OC priming. In osteoimmunological terms, TLR/inflammasome inputs maintain osteoclastogenic tone and couple tumor survival to bone destruction [43,44]. Osteocytes amplify inflammation by modulating RANKL and sclerostin; disruption of perilacunar remodeling alters mechanotransduction and further depresses OB activity [16,45,46,47]. Targeting HMGB1–RAGE may decouple tumor–bone crosstalk and unlock anabolic responses [48].

Collectively, this circuit constitutes an osteo-immunological feed-forward loop that is amenable to therapeutic disruption in combination with antiresorptives, for example by targeting IL-6–driven STAT3 activity or HMGB1–RAGE/TLR engagement in carefully selected patients as demonstrated in breast cancer bone metastasis [49].

Furthermore, soluble IL-6R/gp130–mediated trans-signaling broadens the range of target cells within the marrow and has been implicated in sustaining low-grade inflammatory tone that favors osteoclast priming and osteocyte dysfunction [50,51]. MM cells further secrete CCL3 (MIP-1α) and other chemokines that recruit and polarize monocyte–macrophage progenitors, while BMSCs provide BAFF/APRIL and CXCL12 gradients that stabilize tumor–stromal synapses; together these cues create a permissive, osteoclastogenic cytokine milieu [52,53].

## 5. Autophagy, Proteostasis, and Lysosomal Control

Autophagy plays a fundamental role in supporting osteoclast functionality by coordinating trafficking and polarized secretion of lysosomal components essential for bone resorption. Pioneering work by DeSelm et al. demonstrated that core autophagy-related (ATG) proteins—including Atg5, Atg7, Atg4B, and LC3—are required for correct formation of the ruffled border, the specialized membrane domain through which osteoclasts acidify the extracellular compartment and deliver cathepsin K to the bone matrix [54]. Loss of these ATG factors disrupts targeting of secretory lysosomes to the bone-apposed membrane and impairs both extracellular acidification and matrix digestion, thereby revealing that autophagy machinery orchestrates the polarized exocytic processes underpinning osteoclastic bone resorption. Similar conclusions emerge from independent mechanistic analyses indicating that ATG-dependent membrane dynamics regulate the vectorial transport of lysosomal enzymes and contribute to efficient resorptive activity, reinforcing the view that autophagic pathways are integral to osteoclast secretory competence.

Cytotoxic or metabolic stressors, including chemotherapeutic exposures, profoundly affect osteoclast resorptive output by perturbing the mitochondrial–lysosomal axis, a critical signaling hub that integrates energetic and degradative cues. In particular, transcription factor EB (TFEB)—a master regulator of lysosomal biogenesis and autophagy—is activated in response to mitochondrial ROS generated under stress conditions. Studies in osteoclasts exposed to doxorubicin have shown that mitochondrial ROS oxidize and activate the lysosomal Ca^2+^ channel TRPML1, promoting TFEB nuclear translocation and inducing a compensatory autophagic program. This TFEB/TRPML1-dependent response modulates lysosomal function and alters bone-resorptive behavior, illustrating how stress-responsive lysosomal signaling circuits shape osteoclast adaptation and ultimately determine the magnitude of bone resorption. Additional evidence from broader lysosomal biology further supports the role of TRPML1-mediated Ca^2+^ release in driving TFEB-dependent transcriptional reprogramming under oxidative or metabolic stress, highlighting a conserved mechanism by which lysosomal signaling mitigates organelle dysfunction and restores cellular homeostasis [55].

Proteasome inhibition in MM cells elicits an intense burden on the secretory apparatus, driving accumulation of misfolded immunoglobulin chains and ultimately provoking a terminal unfolded protein response (UPR). This phenomenon reflects the profound proteostasis dependency of MM cells, which rely on both proteasomal and autophagic disposal pathways to maintain viability under high secretory load. Indeed, classical studies have demonstrated that proteasome inhibitors such as bortezomib rapidly activate pro-apoptotic UPR mediators—including PERK, ATF4, and CHOP—leading to a terminal ER-stress response that is tightly intertwined with compensatory autophagic flux in an attempt to mitigate proteotoxic stress.

These findings underscore the integration of proteostasis pathways—including the proteasome, unfolded protein response, and autophagy–lysosomal system—in determining myeloma cell fate. Autophagy demonstrates this integration functionally by buffering proteotoxic stress induced by proteasome inhibition via TFEB-mediated lysosomal expansion, thereby delaying terminal ER-stress–driven apoptosis unless autophagic compensation is specifically disrupted [56,57].

Within this adaptive landscape, TFEB emerges as a master regulator of lysosomal biogenesis and autophagy through its control of the CLEAR (Coordinated Lysosomal Expression and Regulation) network, a genomic program that coordinates the transcription of lysosomal and autophagy-related genes. TFEB activity is dynamically tuned by nutrient and stress cues, primarily via mTORC1-dependent phosphorylation at the lysosomal surface, which retains TFEB in the cytosol under nutrient-replete conditions. In contrast, lysosomal or metabolic stress diminishes mTORC1 activity, enabling TFEB nuclear translocation and transcriptional activation of the CLEAR network, thereby expanding lysosomal capacity and reinforcing autophagic flux. Foundational genomic analyses further show that TFEB directly governs hundreds of CLEAR-network target genes implicated in lysosomal degradation, autophagy, membrane turnover, and broader cellular clearance functions, underscoring its central role in organelle homeostasis [58,59].

**Figure 2:**
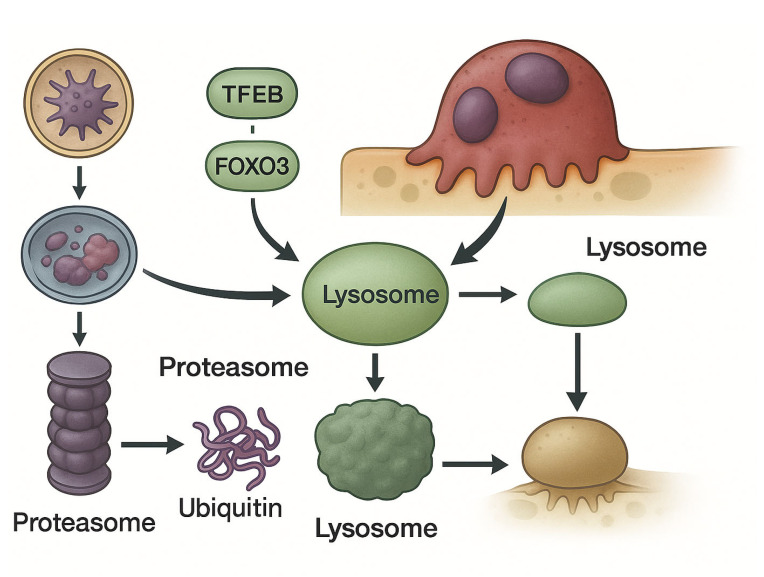
Autophagy–lysosomal remodeling integrates with proteostasis network to regulate osteoclast resorptive activity and myeloma cell survival. In osteoclasts, core autophagy-related (ATG) proteins and lysosomal trafficking coordinate polarized secretion of cathepsin-rich vesicles at the ruffled border, enabling extracellular acidification and bone matrix degradation. Stress-induced mitochondrial ROS activate TRPML1-dependent lysosomal Ca^2+^ release, promoting nuclear translocation of transcription factor EB (TFEB) and induction of the CLEAR network, thereby expanding lysosomal and autophagic capacity. In myeloma cells, proteasome inhibition causes accumulation of misfolded proteins and triggers a terminal unfolded protein response (UPR); compensatory TFEB-dependent autophagy temporarily buffers proteotoxic stress. The functional balance between proteasomal degradation, UPR signaling, and autophagy–lysosomal flux constitutes an integrated proteostasis circuit that determines cell fate and therapeutic sensitivity in the myeloma microenvironment.

In principle, precise and context-specific modulation of TFEB could represent a powerful means to recalibrate cellular metabolism—balancing catabolic and anabolic programs—in a manner sensitive to lineage, differentiation state, and timing. Such targeted manipulation may ultimately permit therapeutic fine-tuning of autophagy-lysosomal pathways in disorders where proteostasis and cellular clearance are critically impaired. See Figure 2.

In myeloma cells, the heavy reliance on proteasome function renders the malignant clone exquisitely sensitive to perturbations in proteostasis, as proteasome inhibition rapidly triggers a maladaptive and ultimately terminal UPR characterized by PERK–ATF4–CHOP activation and ER stress-driven apoptosis. Proteasome blockade is well established to induce compensatory upregulation of autophagy, which serves as a short-term buffering mechanism to mitigate proteotoxic stress. Mechanistically, TFEB is activated by proteasome impairment through dephosphorylation and nuclear translocation, thereby enhancing autophagosome formation and lysosomal function, a response directly demonstrated in proteasome-inhibited cells. The quantitative balance between these proteostasis systems—UPR-driven ER stress and TFEB-governed autophagy—critically shapes therapeutic sensitivity to proteasome inhibitors in multiple myeloma, as synergistic cytotoxicity arises when autophagy buffering is pharmacologically suppressed in the proteasome-inhibited state [60,61,62].

Conceptually, selectively tuning TFEB/CLEAR activity and core ATG components in lineage-specific and temporally restricted ways offers a biologically plausible strategy to restrain osteoclast function while preserving anabolic osteolineage viability. TFEB-driven autophagy is a key determinant of osteolineage homeostasis, with TFEB elevation in osteoblast-lineage cells promoting lysosomal biogenesis, mineralization capacity, and overall bone mass and strength in vivo. In contrast, osteoclast autophagy is required for efficient osteoclast differentiation and bone resorption, and its disruption—such as through impaired lysosomal degradation of TRAF3—reduces osteoclastogenesis and ameliorates myeloma-associated bone loss. Therefore, differential modulation of TFEB-dependent autophagy pathways represents a mechanistically grounded approach to exploit myeloma’s proteostasis addiction while attenuating pathological bone resorption [63,64].

Beyond bulk autophagy, selective modules—mitophagy and ferritinophagy—shape the metabolic and redox set-points that gate osteoclast differentiation. Ferritinophagy couples iron mobilization to lipid peroxidation pressure, intersecting with TFEB-directed lysosomal biogenesis and secretory competence at the ruffled border [65,66,67]. Glucocorticoids, widely used in MM therapy, augment autophagic flux in osteoclasts and can inadvertently potentiate resorptive capacity under inflammatory load, underscoring the need for temporal coordination with antiresorptives or autophagy-modulating strategies [68,69]. Cytotoxic stressors, through mitochondrial ROS and TRPML1-dependent lysosomal calcium, engage TFEB, thereby reprogramming lysosome-centered catabolism and altering resorptive output [70,71,72,73,74].

## 6. Lipid Remodeling, and Ferroptotic Thresholds in Bone and Tumor Compartments

On the lipid axis, ACSL4-dependent enrichment of polyunsaturated phospholipids and the accumulation of oxidized phosphatidylethanolamines under conditions of constrained GPX4 activity define ferroptosis susceptibility [75]. In the marrow niche, these processes establish cell-type-specific redox–lipid rheostats: osteoclast lineage cells experience pro-differentiative iron/ROS signaling, whereas myeloma cells—especially under proteotoxic stress—may be rendered selectively vulnerable to ferroptosis when GPX4 buffering is overwhelmed. Pragmatically, iron modulation (FPN1 restoration or chelation) and context-aware ferroptosis induction represent complementary levers to rebalance remodeling while exerting antitumor pressure [76,77,78,79,80,81].

The ferroptosis/iron-handling corpus expands on ACSL4-driven membrane remodeling, GPX4 constraints, ferritinophagy, and ferroportin biology with direct consequences for osteoclast priming [82].

Importantly, these observations should be interpreted as defining conditional ferroptotic susceptibility rather than deterministic cell fate outcomes. The extent to which ferroptosis contributes to bone remodeling imbalance or tumor control in the clinical setting remains unresolved and is likely to depend on cellular context, disease stage, and concurrent therapeutic pressures.

## 7. Optimizing Early-Phase Assessment of Myeloma Bone Disease Through Standardized Biochemical Markers, Redox Signatures, and Imaging-Based Response Metrics

Among the biomarkers, serum PINP and CTX represent clinically validated, guideline-endorsed measures of bone turnover, whereas redox and iron–lipid peroxidation markers should be regarded as exploratory tools intended for translational research and early-phase trials.

In the context of interventional bone-metabolism trials, the reliability of early pharmacodynamic readouts depends critically on aligning biochemical sampling with imaging-based evaluation windows, thereby maximizing interpretability. Because both PINP and CTX are subject to biologically meaningful fluctuations—particularly circadian oscillations and feeding-dependent suppression of resorption—rigorous pre-analytical standardization is indispensable. Morning, fasted blood draws, and consistent use of validated assay platforms substantially reduce within-subject variability and mitigate artefactual “regression-to-the-mean” effects that can obscure true treatment-related shifts. This principle is firmly supported by consensus recommendations indicating that CTX should always be collected in the early morning in the fasted state, whereas PINP, although more stable, still benefits from harmonized collection conditions to ensure analytic fidelity [82].

When these markers are examined longitudinally as paired deltas across early (baseline → weeks 2–4) and intermediate (weeks 8–12) timepoints, the complementary behavior of formation and resorption markers becomes diagnostically informative. Specifically, an initial CTX decline followed by delayed PINP recovery can differentiate a transient antiresorptive effect from true recoupling of bone remodeling, signaling renewed osteoblastic engagement rather than simple suppression of turnover. Longitudinal PINP/CTX trajectories are well recognized as mechanistically interpretable indicators of evolving treatment response and coupling status in bone-active therapies [83,84].

To refine mechanistic attribution further, concurrent assessment of oxidative and nitrosative stress surrogates—such as advanced oxidation protein products (AOPP), advanced glycation end-products (AGEs), and S-nitrosylated proteins—adds an additional layer of biological resolution. Evidence from both animal and human studies demonstrates that reductions in oxidative burden, including declines in AOPP, precede and predict improvements in bone formation dynamics, thus reinforcing the relevance of redox homeostasis in the normalization of PINP trajectories. Such biomarkers have been repeatedly implicated in the pathophysiology of age-related and postmenopausal bone loss, where oxidative and carbonyl stress directly impair osteoblast–osteoclast coupling and favor resorptive dominance [85].

Finally, explicit control of dietary and renal confounders—both of which can materially influence oxidative load, marker clearance, and turnover signals—is essential for preserving assay sensitivity in early-phase trials. By integrating harmonized sampling, longitudinal Δ-analysis, and redox-state biomarkers, clinical studies can more accurately attribute observed shifts in PINP and CTX to true pharmacologic action rather than to pre-analytical or physiologic noise.

On the imaging axis, timing FDG-PET/CT after early Bone Turnover Markers (BTMs) inflection (e.g., 6–8 weeks) can adjudicate whether biochemical improvements correspond to reductions in metabolically active lesions, whereas WB-MRI and LD-WBCT anchor structural readouts (edema/diffusion changes vs. cortical/trabecular integrity) [86]. Embedding these modalities within IMWG-aligned schedules facilitates concordant hematologic and skeletal response assessment and enables adaptive decisions (intensification vs. de-escalation) in trials testing microenvironment-targeted combinations [87,88]. Finally, exploratory iron–lipid indices (standard iron panels plus research-grade peroxidation surrogates) can be prospectively linked to BTM/imaging composites to enrich for phenotypes predicted to benefit from iron modulation or ferroptosis-aware strategies, while maintaining external validity through guideline-anchored endpoints.

Accordingly, Table 1 summarizes a tiered panel of biomarkers and imaging endpoints, distinguishing clinically established measures from exploratory readouts primarily intended to support mechanism-based trial design rather than routine patient care (Table 1) [89].

## 8. Therapeutic Implications and Roadmap

It is important to clarify that, with the exception of approved antiresorptive agents, most interventions discussed in this section are supported by preclinical, early translational, or indirect clinical evidence derived from related oncologic or inflammatory settings. Accordingly, the therapeutic concepts outlined below should be interpreted as a biologically informed translational framework rather than as evidence-based treatment recommendations for routine clinical practice.

Antiresorptives are foundational in MM Osteolytic Bone Disease (OBD): denosumab is non-inferior to zoledronic acid for Skeletal-Related Event (SRE) prevention and is advantageous in renal impairment [90]. However, antiresorptives alone seldom restore bone formation, necessitating upstream microenvironmental interventions [90]. We therefore propose a three-component therapeutic roadmap explicitly layered on standard antiresorptive care.

**Figure 3:**
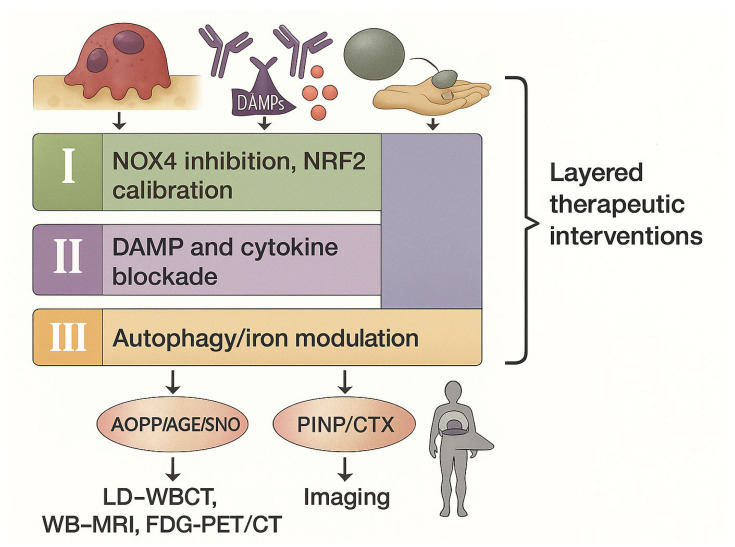
Layered therapeutic strategies targeting microenvironmental stress programs to restore coupled bone remodeling in multiple myeloma. Standard antiresorptive therapy (e.g., RANKL inhibition) forms the backbone for skeletal protection but is insufficient to restore bone formation. Upstream redox reprogramming through selective NOX4 inhibition and calibrated NRF2 activation reduces pathological ROS signaling while preserving physiological redox functions. Parallel anti-inflammatory interventions targeting DAMP–RAGE/TLR pathways and cytokine axes (IL-1β, TNF-α, IL-6) decompress chronic osteoimmune activation. Lineage- and timing-specific modulation of autophagy and iron handling—including TFEB-dependent lysosomal tuning, ferroportin-1 restoration, or iron chelation—restrains osteoclast resorptive capacity while protecting osteoblast viability and, in selected contexts, sensitizing myeloma cells to ferroptotic stress. Integration of these interventions with standardized biochemical markers (PINP/CTX), redox biomarkers, and IMWG-endorsed imaging provides a framework to monitor true recoupling and structural bone regeneration.

(i) Redox reprogramming with selective NOX4 inhibition and calibrated NRF2 to blunt pathological ROS while preserving host defenses. (ii) Anti-inflammatory co-targeting to disrupt DAMP–RAGE/TLR and IL-1β/TNF-α/IL-6 circuits, thereby normalizing OC priming and enabling anabolic recovery. (iii) Autophagy/iron modulation—lineage- and timing-specific autophagy tuning to protect osteolineage while exploiting myeloma proteostasis addiction; restoration of FPN1 or iron chelation to limit osteoclastogenesis and adjust ferroptosis thresholds. See Figure 3.

It should be emphasized that therapeutic strategies aimed at modulating ferroptosis or iron–lipid peroxidation pathways are, at present, primarily supported by preclinical and early translational evidence. As such, they should be viewed as hypothesis-generating frameworks rather than established clinical interventions, pending prospective validation in appropriately designed clinical trials.

### Operationalization and Sequencing

In practical terms, the proposed therapeutic roadmap can be operationalized through a phased and biomarker-guided sequencing strategy, in which representative pharmacologic agents are aligned with dominant microenvironmental stress programs while preserving physiological bone remodeling.

**Phase A (Stabilization)** is centered on the rapid attenuation of pathological osteoclastogenic signaling and is explicitly layered on a standard antiresorptive backbone (e.g., denosumab or zoledronic acid). At this stage, early redox reprogramming may be introduced using selective NOX4 inhibition—exemplified by agents such as setanaxib (GKT137831), which has been extensively characterized as redox-driven inflammatory and fibrotic pathologies—as a prototype for dampening pathological ROS flux while sparing constitutive NADPH oxidase activity. In parallel, calibrated NRF2 modulation may be achieved through indirect or moderate activators (e.g., dimethyl fumarate or nutraceutical-derived NRF2 inducers such as sulforaphane), explicitly avoiding supraphysiologic pathway activation that could impair immune surveillance or osteoblast differentiation. The intent of this phase is not anabolic induction but rapid narrowing of the resorptive–oxidative window, as evidenced by early declines in CTX and oxidative stress composites [33,91,92,93].

**Phase B (Decompression)** targets the sterile inflammatory circuits that sustain osteoclast priming once resorptive dominance has been stabilized. In patients displaying high inflammatory signatures—defined by elevated AOPP/AGEs, cytokine profiles, or FDG-PET/CT avidity—this phase incorporates targeted anti-inflammatory cotreatments selected to map onto specific mechanistic axes. Representative examples include IL-6 pathway inhibition (e.g., tocilizumab as a biologic precedent for STAT3-driven osteoimmune amplification), IL-1β blockade (e.g., anakinra or canakinumab), and experimental approaches aimed at HMGB1–RAGE or TLR signaling disruption, which are supported by preclinical and translational oncology data. Importantly, these interventions are envisioned as temporally bounded and biomarker-guided, aimed at decompressing cytokine pressure rather than imposing chronic immunosuppression, thereby restoring a permissive environment for osteolineage recovery [94,95,96,97].

**Phase C (Recoupling)** is initiated only after biochemical evidence of resorptive slowing (CTX suppression with early PINP stabilization) and focuses on restoring formation while preserving lineage viability. At this stage, lineage- and timing-specific modulation of autophagy–lysosomal function may be introduced, with clinically available lysosome-targeting agents such as hydroxychloroquine serving as representative examples of autophagy flux modulation, and upstream regulators (e.g., mTORC1–TFEB axis) considered at a conceptual level. In parallel, iron-handling interventions—including iron chelation (e.g., deferasirox) or experimental modulation of the hepcidin–ferroportin axis—can be synchronized with proteasome-based antimyeloma regimens to restrain osteoclastogenesis while exploiting differential ferroptotic thresholds in tumor cells. Critically, this phase is designed to favor true remodeling recoupling rather than prolonged turnover suppression, with PINP recovery and imaging-based structural consolidation as primary readouts [54,98,99].

At present, this phase remains exploratory and hypothesis-generating, and its clinical feasibility and safety will need to be established in prospective, biomarker-driven clinical trials specifically designed to assess skeletal outcomes.

Across all phases, adaptive sequencing keyed to early shifts in PINP/CTX trajectories, oxidative/inflammatory composites, and IMWG-endorsed multimodal imaging allows iterative refinement of dose and timing within weeks, rather than months. Safety considerations must remain lineage-resolved: surveillance of cortical microarchitecture and osteocyte integrity mitigates the risk of over suppressing physiological remodeling, while parallel immune and hematologic monitoring preserves systemic robustness. Collectively, this staged operationalization translates control of redox, inflammatory, and autophagy–iron axes into a clinically actionable framework aimed not merely at limiting bone loss, but at re-establishing coupled bone remodeling and durable skeletal recovery in multiple myeloma.

## 9. In Vivo Models of Multiple Myeloma Bone Disease Supporting Redox, Inflammatory, and Autophagy–Iron Pathways

Reliable in vivo models of MM are essential to validate the causal role of microenvironmental stress-adaptation programs—including oxidative stress, sterile inflammation, autophagy–lysosomal remodeling, and iron-dependent lipid peroxidation—in the pathogenesis of osteolytic bone disease. Murine systems have provided robust experimental platforms to interrogate these pathways under physiological bone remodeling constraints and immune contexture.

### 9.1. Syngeneic Immunocompetent Models

The 5T family of murine myeloma models (notably 5T2MM and 5T33MM), generated by serial transplantation of spontaneously arising myeloma cells into syngeneic C57BL/KaLwRij mice, remains the gold standard for studying MM-associated bone disease in an immunocompetent setting. These models faithfully recapitulate hallmark features of human disease, including bone-restricted plasma cell growth, osteoclast-driven osteolysis, increased marrow angiogenesis, and inflammatory cytokine production, making them particularly suitable for evaluating osteoimmune and redox-dependent mechanisms. Crucially, 5T models have been extensively used to demonstrate in vivo roles of RANKL excess, IL-6 signaling, oxidative stress, and osteoprotegerin imbalance, as well as to test antiresorptive and microenvironment-targeted therapies [100].

### 9.2. Human Xenograft and Intrabone Models

Human MM xenografts—derived from commonly used cell lines such as MM.1S, RPMI-8226, and U266—implanted into immunodeficient hosts (SCID or NSG mice) provide complementary systems to dissect tumor-intrinsic stress responses. When delivered via intrabone or systemic intravenous injection, these models reproduce localized osteolytic lesions, marrow hypoxia, and proteostasis stress, allowing mechanistic interrogation of autophagy dependence, redox imbalance, and ferroptosis susceptibility under controlled genetic backgrounds. Such xenografts have been instrumental in validating the contribution of oxidative stress and hypoxic signaling to MM progression and bone destruction in vivo [101].

### 9.3. Genetically Engineered and Pathway-Focused Mouse Models

Beyond tumor-bearing systems, genetically modified mice have been essential for defining cell-type-specific roles of stress pathways implicated in MM bone disease. Conditional modulation of NOX4, NRF2, or key autophagy-related genes (*ATG5, ATG7, TFEB*) in osteoclast or osteoblast lineages has demonstrated that redox-driven autophagy and lysosomal function directly regulate osteoclast differentiation, resorptive capacity, and coupling signals. In particular, NOX4-dependent ROS production has been shown to promote osteoclastogenesis through autophagy-linked stress pathways, providing mechanistic validation for selective redox targeting in vivo [102].

### 9.4. Iron Handling and Ferroptosis-Related Models

Emerging in vivo evidence implicates iron metabolism and ferroptosis thresholds as modulators of both tumor survival and osteoclast priming in MM. Mouse models integrating myeloma growth with manipulation of ferroportin, ferritinophagy, or GPX4-associated lipid peroxidation have demonstrated that iron retention amplifies osteoclast activity while simultaneously sensitizing myeloma cells to ferroptotic stress. Recent studies using genetically defined myeloma models and stromal cell-conditioned niches confirm that bone marrow microenvironmental iron handling dictates ferroptosis susceptibility in vivo, highlighting a dual tumor–bone regulatory axis [103].

Collectively, these in vivo murine systems provide convergent validation that oxidative, inflammatory, autophagy-lysosomal, and iron-lipid peroxidation pathways are not merely associative but causally linked to pathological bone remodeling in MM. The use of standardized endpoints—including micro-CT, histomorphometry, serum PINP/CTX, and molecular imaging—closely parallels IMWG-recommended clinical assessments, reinforcing the translational bridge between preclinical modeling and therapeutic development [104] (Table 2).

## 10. Translational Outlook

Multiple myeloma–related bone disease can be understood as the convergence of two tiers of pathobiology. At the foundational level, canonical remodeling imbalances—excessive RANKL signaling in the face of inadequate OPG and a parallel blockade of the Wnt pathway—tip the remodeling balance toward unchecked osteoclast activity and suppressed osteoblastogenesis. Superimposed upon this are three higher-order stress programs—oxidative stress, sterile inflammation, and autophagy–lysosomal remodeling—that do not merely coexist with canonical defects but stabilize them over time, creating a self-reinforcing niche that favors osteolysis and impedes structural repair. From a translational standpoint, modern IMWG-endorsed imaging and standardized bone-turnover markers provide a common language to quantify both structural injury (extent and quality of trabecular and cortical loss) and metabolic marrow activity (lesion avidity and residual disease), thereby enabling trials to track the osteolytic process and its modification under therapy with greater fidelity. Together, this integrated framework explains why skeletal lesions often fail to consolidate despite hematologic control and clarifies which upstream nodes should be prioritized when the goal is not merely to flatten resorption but to recouple remodeling and deliver clinically meaningful skeletal recovery [6,76,89].

Importantly, this hierarchical organization carries direct translational implications. Interventions targeting upstream stress-amplifying programs—such as oxidative and inflammatory signaling—are predicted to exert broader and more durable effects on bone remodeling than strategies aimed at downstream vulnerabilities alone, including ferroptosis modulation, which remains context-dependent and incompletely validated in vivo.

## 11. Conclusions

As reported above, two interconnected levels of pathobiology come together to cause bone damage associated with multiple myeloma. The uncoupling of bone resorption and formation is initiated at the foundational tier by classical remodeling imbalance, which is caused by excessive RANKL signaling, insufficient osteoprotegerin buffering, and persistent suppression of Wnt-dependent osteoblastogenesis. Microenvironmental stress-adaptation programs actively stabilize and intensify the uncoupled condition on top of this structural flaw.

A self-reinforcing network that reduces bone formation and increases osteoclast competency is formed by the convergence of oxidative stress, sterile inflammation, and autophagy–lysosomal remodeling. In addition to acting as effectors, reactive oxygen species also serve as signaling amplifiers that enhance RANKL/NFATc1-driven osteoclastogenesis, make stromal and osteocytic compartments more sensitive to inflammatory signals, and discourage mesenchymal progenitors from committing to osteoblasts. Anabolic osteolineage pathways are directly inhibited while activation thresholds in osteoclast precursors are lowered by DAMP-driven inflammatory signaling via RAGE/TLR and cytokine circuits. This mismatch is further reinforced by autophagy–lysosomal remodeling, which is controlled by TFEB/CLEAR signaling and supports osteoclast secretory capacity under stress while placing context-dependent restrictions on osteoblast differentiation and mineralization.

Crucially, these stress programs create a feed-forward system that keeps bone remodeling in a chronically resorptive, non-regenerative state by mechanistically integrating with conventional remodeling errors rather than just coexisting with them. This concept explains why antiresorptive treatments alone seldom restore bone formation and why skeletal lesions often fail to heal despite excellent hematologic management.

From a translational perspective, this synthesis finds practical upstream nodes that might be used to restore linked remodeling. Through context-aware NRF2 modulation and specific NOX4 suppression, calibrated redox reprogramming can relax osteoclastogenic signals while maintaining physiological redox functions necessary for bone production. Osteoblast re-engagement may be made possible by the disruption of cytokine-driven inflammatory pathways and DAMP-RAGE/TLR. Lastly, controlling autophagy and iron management according to lineage and timing provides a way to limit pathological osteoclast activity while preserving osteolineage viability and, in some situations, taking advantage of variable ferroptotic thresholds in tumor cells.

Harmonized evaluation methods, such as coupled PINP/CTX trajectories, redox and inflammatory biomarkers, and multimodal IMWG-endorsed imaging to differentiate real recoupling from simple turnover suppression, are necessary to operationalize these principles.

To restore balanced bone remodeling and achieve clinically meaningful skeletal regeneration in multiple myeloma, it makes sense to integrate microenvironment-targeted strategies with standard anti-myeloma therapy.

## Figures and Tables

**Table 1:** Biomarkers and imaging endpoints for trial design.

Domain	Measure	Rationale	Assay/Acquisition Notes	Endpoints
Bone turnover	s-PINP; s-CTX	Standardized monitoring of remodeling dynamics	Follow IOF–IFCC preanalytics and variability control	Primary PD; correlate with imaging/fractures
Redox status	AOPP; AGEs; S-nitrosylated proteins	Microenvironmental oxidative/nitrosative load	Batch controls; diet/renal confounders	Secondary PD; link to NOX4/NRF2 strategies
Imaging	LD-WBCT; WB-MRI; FDG-PET/CT	Structural + metabolic assessment	IMWG protocols; harmonize PET/CT timing	Structural healing; metabolic remission; SREs
Iron–lipid peroxidation	Iron indices; lipid peroxidation markers	Infer ferroptosis/OC iron handling	Standard iron panel + research lipidomics	Exploratory PD; responder enrichment

**Table 2:** Representative murine models supporting redox, inflammatory, autophagy–lysosomal, and iron–lipid peroxidation pathways in multiple myeloma–associated bone disease. The models listed have been widely used for mechanistic validation and translational testing.

Model	Host Immune Status	Key Pathways Validated In Vivo	Translational/Therapeutic Relevance
5T2MM/5T33MM	Syngeneic, immunocompetent	RANKL–OPG imbalance; IL-6–driven inflammation; oxidative stress; osteoclastogenesis	Validation of antiresorptives; cytokine modulation; redox-targeted strategies
MM.1S intrabone xenograft	SCID/NSG	Proteostasis stress; hypoxia; autophagy dependence; oxidative signaling	Proteasome–autophagy co-targeting; stress-response pathway inhibition
U266/RPMI-8226 xenografts	Immunodeficient	Redox imbalance; inflammatory cytokine signaling	Tumor-intrinsic redox and cytokine pathway targeting
Conditional NOX4/ATG/TFEB mice	Immunocompetent, lineage-specific	Redox–autophagy–lysosomal coupling in osteoclasts and osteoblasts	Selective NOX4 inhibition; lineage-aware autophagy modulation
Ferroptosis-related MM models	Syngeneic or xenograft (context-dependent)	Iron retention; lipid peroxidation; ferroptotic thresholds	Iron modulation; ferroptosis-inducing strategies

## Data Availability

Not applicable.

## Abbreviations

MAPK	Mitogen-Activated Protein Kinase
IOF	International Osteoporosis Foundation
IMWG	International Myeloma Working Group
TLR	Toll-Like Receptor
CLEAR	Coordinated Lysosomal Expression and Regulation network
DHODH	Dihydroorotate Dehydrogenase
WNT	Wingless-type Signaling Pathway
RANK	Receptor Activator of Nuclear Factor-κB
IFCC	International Federation of Clinical Chemistry
MM	Multiple Myeloma
NADPH	Nicotinamide Adenine Dinucleotide Phosphate
OB	Osteoblast
WBCT	Whole-Body Computed Tomography
BTM/BTMs	Bone Turnover Marker(s)
LD-WBCT	Low-Dose Whole-Body CT
ER	Endoplasmic Reticulum
EB	Earliest Binding
MDR	Multidrug Resistance
PRR	Pattern-Recognition Receptor
BAFF	B-cell Activating Factor
MRI	Magnetic Resonance Imaging
OC	Osteoclast
CREB	cAMP Response Element-Binding protein
FDG	Fluorodeoxyglucose
ROS	Reactive Oxygen Species
CTX	C-Terminal Telopeptide of Type I Collagen
OBD	Osteolytic Bone Disease
CLL	Chronic Lymphocytic Leukemia
SRE	Skeletal-Related Event
MIP (MIP-1α)	Macrophage Inflammatory Protein-1 alpha
PET	Positron Emission Tomography
NOX/NOX4	NADPH Oxidase/NADPH Oxidase 4
MRD	Minimal Residual Disease
CT	Computed Tomography
PINP	Procollagen Type I N-Terminal Propeptide
TFEB	Transcription Factor EB
RAGE	Receptor for Advanced Glycation End-products
APRIL	A Proliferation-Inducing Ligand
AOPP	Advanced Oxidation Protein Products
WB	Whole Body
DAMP	Damage-Associated Molecular Pattern
RANKL	Receptor Activator of NF-κB Ligand
OPG	Osteoprotegerin
NF (NF-κB)	Nuclear Factor Kappa-Light-Chain-Enhancer of Activated B Cells
TGF (TGF-β)	Transforming Growth Factor-β
TNF (TNF-α)	Tumor Necrosis Factor-α
IL (IL-1β, IL-6 ecc.)	Interleukin
ATG	Autophagy-Related Gene/Protein
